# Visits to Private Asylums

**Published:** 1903-08-22

**Authors:** 


### VISITS TO PRIVATE ASYLUMS.

By Our Rambling Reporter.

### CLAYBURY HALL, WOODFORD, ESSEX.

When the estate of Claybury, near Woodford Bridge, was acquired for the purpose of building thereon a large county asylum for London, the authorities very wisely decided to retain the fine old Georgian Hall, and to fit it up as a residence for private patients. The total amount of land runs to about three hundred acres, and several acres immediately surrounding the old hall have been kept and laid out as pleasure grounds for these private patients. There is much old timber about, and the hall is not only well protected from the north winds, but it is screened from the large buildings which constitute the asylum proper, and which from this standpoint are but little in evidence.

To obtain the requisite amount of sleeping accommodation a new wing had to be added, but we were glad to note that the house itself had been very little interfered with; and we can still wander about the handsome, well-proportioned rooms and halls, with their Flaxman and Angelica decorations, scarcely altered from what they were in those Georgian times when they served a different if less useful purpose.

All these old rooms are light and airy, and suitably furnished. Some of them command fine views of the valley lying to the south, and far off we can discern Shooter's Hill, with a silver streak between, which we are told is the river Thames, as it flashes in the sun on this bright day in spring.

All the rooms in the hall are now lighted by electricity. The dormitories are well ventilated, and the separate bedrooms are of good size, and well furnished.

Altogether the hall will accommodate 50 patients, and only men are received. These private patients are under the immediate care of a highly qualified resident medical officer, who evinces the greatest interest in his asylum work; and, of course, the medical superintendent is at hand with all his varied and immense experience in the treatment of mental diseases. The friends of the patients may therefore feel the utmost confidence that everything is within reach that can lead to the recovery of the patient, or to his comfort when cure is impossible.

The charge for London patients is 30s. a week, and it cannot be denied that the accommodation provided, and the attention given to the patient, is worth this sum or even more from a market value standpoint; nevertheless, we should like to learn that the committee could see their way to reduce the amount to £1 in some cases at least, as we feel sure that such a reduction would meet the exigencies of a numerous and deserving class. To this class the difference between £78 a year and £52, is a very real and important one.

It is to us a never-failing source of wonder and regret that every county in England has not provided a block for the treatment of private patients within the grounds of the county asylums.

